# Numerical Sensing of Plastic Hinge Regions in Concrete Beams with Hybrid (FRP and Steel) Bars

**DOI:** 10.3390/s18103255

**Published:** 2018-09-27

**Authors:** Fang Yuan, Mengcheng Chen

**Affiliations:** School of Civil Engineering and Architecture, East China Jiaotong University, Nanchang 330013, China; mcchen@ecjtu.edu.cn

**Keywords:** plastic hinge length, hybrid reinforced, concrete beam, fibre-reinforced polymer, steel, finite element analysis

## Abstract

Fibre-reinforced polymer (FRP)-reinforced concrete members exhibit low ductility due to the linear-elastic behaviour of FRP materials. Concrete members reinforced by hybrid FRP–steel bars can improve strength and ductility simultaneously. In this study, the plastic hinge problem of hybrid FRP–steel reinforced concrete beams was numerically assessed through finite element analysis (FEA). Firstly, a finite element model was proposed to validate the numerical method by comparing the simulation results with the test results. Then, three plastic hinge regions—the rebar yielding zone, concrete crushing zone, and curvature localisation zone—of the hybrid reinforced concrete beams were analysed in detail. Finally, the effects of the main parameters, including the beam aspect ratio, concrete grade, steel yield strength, steel reinforcement ratio, steel hardening modulus, and FRP elastic modulus on the lengths of the three plastic zones, were systematically evaluated through parametric studies. It is determined that the hybrid reinforcement ratio exerts a significant effect on the plastic hinge lengths. The larger the hybrid reinforcement ratio, the larger is the extent of the rebar yielding zone and curvature localisation zone. It is also determined that the beam aspect ratio, concrete compressive strength, and steel hardening ratio exert significant positive effects on the length of the rebar yielding zone.

## 1. Introduction

The corrosion of steel reinforcement ultimately causes the loss of serviceability or the strength failure of reinforced concrete (RC) structures. Noncorrosive fibre-reinforced polymer (FRP) is an alternative to steel reinforcement for concrete structures [1,2,3,4,5,6,7,8,9]. However, the linear-elastic property of FRP rebars results in the brittle structural behaviour of FRP-reinforced concrete structures. The design of the FRP-reinforced concrete flexural member must be aimed at achieving the concrete compression failure mode [10,11,12,13]. By experiencing concrete crushing prior to the tensile rupture of the FRP reinforcement, a flexural member exhibits a particular limited inelastic behaviour prior to failure.

Alternatively, the structural performance of concrete beams can be improved by a combination of FRP and steel reinforcements. An improved durability compared with RC beams is also likely to be obtained by placing FRP rebars at the corners or near the extreme tension face [14]. Multiple tests have been conducted on hybrid FRP–steel reinforced concrete beams [14,15,16,17,18,19,20,21,22]. It has been reported that the strength and ductility can be improved simultaneously by appropriately designing the hybrid reinforcement ratio between FRP and steel [23]. Current research on hybrid reinforced beams focuses on crack-width calculation, deflection evaluation, or strength prediction; few studies concentrate on plastic hinge problems. Nevertheless, the length of the plastic hinge region is critical for structural retrofit and design [24]. On the one hand, the plastic hinge length determines the retrofitting area. On the other hand, designing new structures also requires this knowledge in order to predict the deformation capacity. This necessitates an extensive study of the plastic hinge problem of hybrid FRP–steel reinforced members.

Numerous tests have been carried out on the plastic hinge of RC members, and many plastic hinge length models have been proposed [25,26,27,28,29,30,31,32,33,34]. Recently, three plastic hinge regions—the rebar yielding zone, concrete crushing zone, and curvature localisation zone—have been proposed, in order to discuss the plastic hinge problems of RC members [24] and FRP-reinforced concrete members [35,36,37]. It was determined that the concrete crushing length governs the seismic retrofitting region, whereas the length of the curvature localisation zone determines the ultimate deformation.

This study is an attempt to assess the plastic hinge problem of hybrid reinforced beams through finite element analysis (FEA). Firstly, a finite element model is proposed, so as to validate the accuracy of the numerical method. The effect of the hybrid reinforcement ratio on the lengths of the three plastic hinge regions is analysed in detail. Subsequently, the influence of the main parameters on the plastic hinge lengths of hybrid reinforced beams is evaluated through parametric studies.

## 2. Finite Element Modelling and Implementation

The general finite element software ABAQUS [38] is used in this work. The constitutive models involve concrete, steel reinforcement, FRP reinforcement, and interfaces between concrete–steel and concrete–FRP. Detailed modeling as well as the parameters are summarised below.

### 2.1. Modelling of Concrete

The four-node linear plane stress quadrilateral element CPS4R in ABAQUS is employed for the concrete elements. The mesh size of the concrete element is 20 mm. Elements smaller than 20 mm make little difference to the results, according to the mesh convergence model.

**Figure 1:**
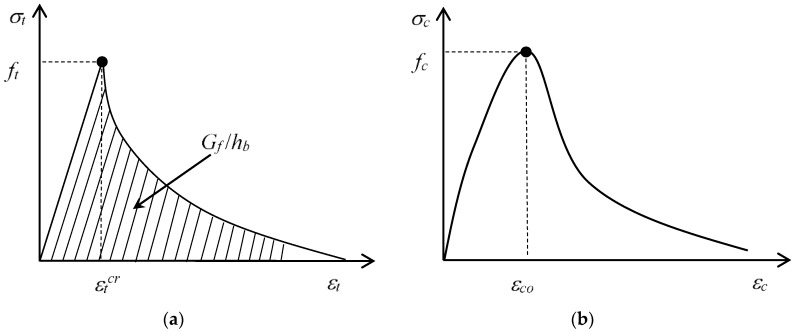
Stress–strain relationship of concrete: (**a**) tension model; (**b**) compression model.

The damaged plasticity model is adopted to model the material behavior of concrete. The ascending section of the tensile relationship is assumed to be linear, and the softening is assumed to be exponential, as shown in Figure 1a, where *f_t_* refers to the tensile strength; $\varepsilon_{cr}^{t}$ indicates the strain at peak stress; *G_f_* is the fracture energy of concrete; and *h_b_* is the crack band width and was taken to be $\sqrt{2A}$, where *A* is the total area of the element [39]. The fracture energy method is adopted for the post-peak cracking of concrete. In the present work, $\varepsilon_{cr}^{t}$ is assumed to be 0.0001 and *G_f_* is expressed by the following:

(1) $$G_{f}=\left(0.0469d_{a}^{2}-0.5d_{a}+26\right){\left(\frac{f_{co}}{10}\right)}^{0.7}$$

where *f_co_* is the concrete compressive strength, and *d_a_* is the maximum size of the coarse aggregate and is considered to be 20 mm in this work.

For concrete under compression, the widely used stress–strain model proposed by Popvics [40] is used in the present study (Figure 1b), as follows:

(2) $$\sigma_{c}=\frac{f_{co}xr}{r-1+x^{r}}$$

in which

(3) $$x=\frac{\varepsilon }{\varepsilon_{co}}$$

(4) $$r=\frac{E_{c}}{E_{c}-f_{co}/\varepsilon_{co}}$$

(5) $$\varepsilon_{co}=9.37\times 10^{-4}f_{co}^{0.25}$$

(6) $$r=\frac{E_{c}}{E_{c}-f_{co}/\varepsilon_{co}}$$

(7) $$E_{c}=4730\sqrt{f_{co}}$$

where *σ_c_* and *ε_c_* are the compressive stress and strain of concrete, respectively; *f_co_* is the peak stress; *ε_co_* is the strain at *f_co_*; and *E_c_* is the elastic modulus of concrete.

Poisson’s ratio is assumed to be 0.2 and the dilation angle is selected to be 55° for concrete. The following default plastic parameters are used for concrete in ABAQUS: 0.1 for eccentricity, 1.16 for the ratio of the initial equi-biaxial compressive yield stress to the initial uniaxial compressive stress, and 0.6667 for the ratio of the second stress invariant on the tensile meridian to that on the compressive meridian.

### 2.2. Modelling of Reinforcement

**Figure 2:**
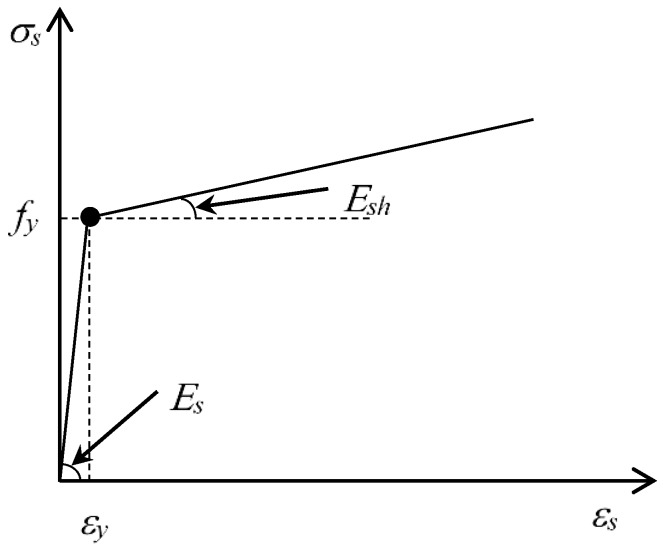
Stress–strain relationship of steel reinforcement.

A two-node truss element, T3D2 in ABAQUS, is employed for the steel reinforcement and FRP reinforcement. The bilinear model, which considers strain hardening, is adopted to model the stress–strain relationship of the steel reinforcement (Figure 2). The stress–strain relationship can be expressed as follows:

(8) $$\{\begin{array}{cc} \sigma_{s}=E_{s}\varepsilon_{s} & 0\leq \varepsilon_{s}\leq \varepsilon_{y}\\ \sigma_{s}=f_{y}+E_{sh}\left(\varepsilon_{s}-\varepsilon_{y}\right) & \varepsilon_{s}>\varepsilon_{y} \end{array}$$

where *σ_s_* and *ɛ_s_* are the stress and strain of steel, respectively; *ɛ_y_* is the yield strain of steel; *E_s_* is the elastic modulus of steel; and *E_sh_* is the hardening modulus of steel. FRP reinforcement is treated as a linear-elastic material, where the stress increases linearly with the increasing strain.

### 2.3. Bond–Slip Relationship

The Spring2 element is used to define the nonlinear bond–slip relationship between steel/FRP reinforcements and concrete. The reinforcement nodes are connected with concrete nodes by a series of springs. A very large stiffness is employed for the springs in the normal direction of the interface, to ensure the same deformation in the direction of the reinforcements and the concrete. In the tangential direction, the bond–slip relationship proposed by Wu and Zhao [41] is employed to consider the nonlinear slip between the concrete and longitudinal reinforcement. The bond stress–slip relationship can be transformed into the nonlinear spring force–relative displacement relationship by the following:

(9) $$F=\tau \cdot \pi \cdot d_{b}l_{s}$$

where *τ* is the bond stress, and *l_s_* is the distance between the spring elements, which is the same as the mesh size of 20 mm. It is noted that the nonlinear springs are used only for longitudinal reinforcement. The transverse reinforcement is treated as the embedded elements in ABAQUS. For more details, please refer to the previous literature by the first author [42,43].

## 3. Model Verification

**Figure 3:**
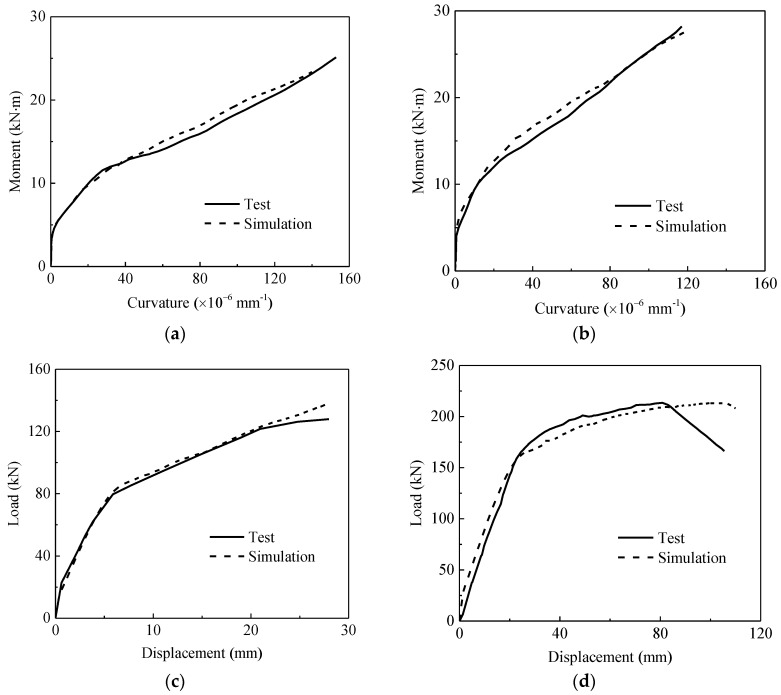
Load–deformation curves: (**a**) A1, (**b**) A2, (**c**) B3, and (**d**) G0.6-T1.0-A90.

Four concrete beams reinforced with hybrid FRP–steel bars are simulated. Table 1 presents details of the beams [19,21]. Specimen A1 and G0.6-T1.0-A90 exhibit steel reinforcement ratios higher than the FRP reinforcement ratio, whereas specimens A2 and B3 exhibit FRP reinforcement ratios higher than the steel reinforcement ratio. Figure 3 shows the load–deformation curves of the specimens. The simulated ultimate curvature of specimens A1 and A2 are 8.42% lower and 2.63% higher, respectively, than the measured results, and the simulated load carrying capacity of specimen B3 and G0.6-T1.0-A90 are 8.35% higher and 0.04% lower, respectively, than the measured ones.

## 4. Plastic Hinge Analysis of Hybrid FRP–Steel Reinforced Beams

**Figure 4:**
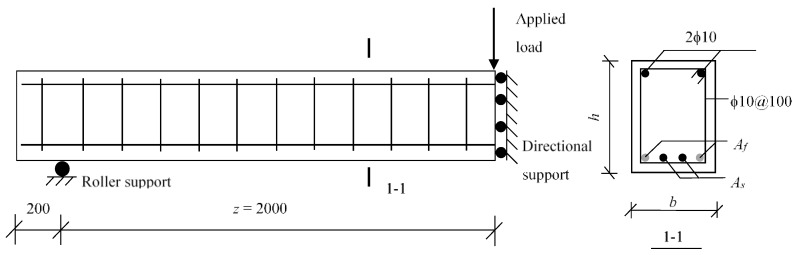
Details of control beam (unit: mm).

This section describes the analysis of the influence of the FRP reinforcement ratio on the development of the plastic hinge regions for hybrid FRP–steel reinforced concrete beams. Four hybrid reinforced beams with identical total reinforcement areas, albeit different hybrid reinforcement ratios, are selected for the analysis. The hybrid reinforcement ratio defined by Qin et al. [23] is used in the present study, and it is expressed by the following:

(10) $$\rho_{r}=\frac{A_{f}}{A_{s}}$$

where *A_f_* and *A_s_* are the reinforcement areas of FRP and steel, respectively. Herein, the total tension reinforcement ratio is fixed to be 1.6% of the four beams. The FRP reinforcement ratios are selected as 0, 0.4%, 0.8%, and 1.2%, corresponding to the hybrid reinforcement ratios of 0, 0.33, 1, and 3, respectively. All of the specimens exhibit identical geometric dimensions and material properties. A typical cantilever beam is investigated in the present study, as shown in Figure 4. The specimens have a cross-section of *b* × *h* = 200 mm × 400 mm. The distance from the middle section to the support point (*z*) is 2000 mm (Figure 4). The material properties of the steel reinforcement are as follows: yield strength *f_y_* = 460 MPa, ultimate strength *f_u_* = 610 MPa, elastic modulus *E_s_* = 200 GPa, and hardening modulus to elastic modulus ratio *E_sh_*/*E_s_* = 0.01. The tensile properties of CFRP (Carbon fiber-reinforced polymer) reinforcement are as follows: elastic modulus *E_f_* = 160 GPa and ultimate strength *f_fu_* = 2400 MPa. The concrete compressive strength *f_co_* = 30 MPa.

### 4.1. Investigation of Rebar Yielding Zone

**Figure 5:**
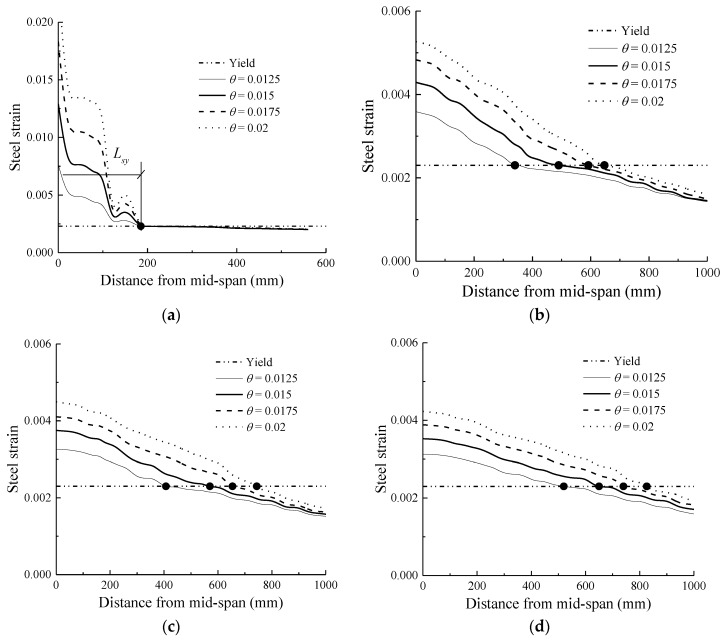
Longitudinal steel strain distribution: (**a**) *ρr* = 0; (**b**) *ρr* = 0.33; (**c**) *ρr* = 1; (**d**) *ρr* = 3.

For a beam reinforced only by steel (*ρ_r_* = 0), an evident dividing point exists in the steel strain distribution curves. The rebar strains on the left side of the dividing point attain or exceed the yield strain and grow dramatically with an increase in the drift ratio. However, on the right side of the dividing point, the rebar strains are lower than the yield strain and remain almost constant as the drift ratio increases, as shown by the longitudinal rebar strain distributions for the specimens at each drift ratio level in Figure 5. These simulation results are consistent with the test observations [44,45]. In the present study, the distance from the mid-span section to the dividing point is defined as the rebar yielding length (*L_sy_*), as shown in Figure 5a.

**Figure 6:**
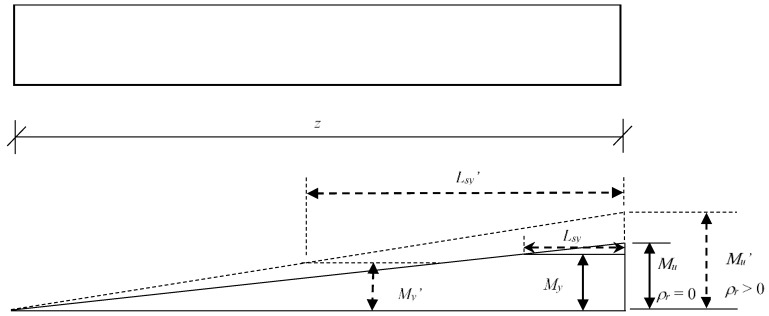
Schematic diagram of moment distributions for cases of *ρr* = 0 and *ρr* > 0.

The steel strain variation trend of the hybrid FRP–steel reinforced concrete beams are completely different from that of the RC beam, as shown in Figure 5b–d. It is evident from these figures that the steel strains continue to increase as the drift ratio increases. As a result, no fixed dividing point exists, and *L_sy_* continues to increase as the drift ratio increases. This can be explained as follows. For the normal RC beam (*ρ_r_* = 0), the difference between the maximum moment and the yield moment remains constant after the load carrying capacity is attained. In this case, the distance from the yield section to the maximum moment section (mid-span section in this study), which is known as *L_sy_*, remains constant after a specific displacement. However, for the hybrid reinforced concrete beams (*ρ_r_* > 0), the yield moment occurs at a fixed displacement, whereas the moment resistance continues to increase, owing to the linear-elastic property of the FRP reinforcement. In this case, the gap between the maximum moment and the yield moment continues to increase as the drift ratio increases, resulting in an increasing *L_sy_*. The higher the hybrid reinforcement ratio, *ρ_r_*, the more significant the increment of *L_sy_*. On the one hand, the yield moment decreases with the increasing *ρ_r_*, owing to the smaller steel reinforcement layout. On the other hand, the maximum moment continues to increase as *ρ_r_* increases, owing to the higher tensile strength of the FRP reinforcement compared with that of the steel reinforcement. As a result, the gap between the mid-span moment and the yield moment grows larger, as illustrated in Figure 6. For example, *L_sy_* increases by 265% at a drift ratio of 2% when *ρ_r_* changes from zero to 0.33, compared with 308% and 351% when *ρ_r_* varies from zero to one and three, respectively [46,47].

RC with hybrid FRP–steel rebars guarantees a greater ultimate deformability and deflection than RC with only steel rebars, but it does not guarantee more ductility. The reason the ultimate displacement of the former is greater than that of the latter has not been explained by the authors, which is a serious flaw. The reason is that when the behavior of the steel rebars is plastic, the behavior of the FRP rebars is linear-elastic, so the inelastic part of the moment–curvature diagram is a steep curve up to the final level. On the contrary, the inelastic part of the moment–curvature diagram of a steel section is flat. Therefore, in the former case, the length of the plastic hinge is greater than in the latter case, and the ultimate displacement is also greater. Nevertheless, that behavior consists of deformability not ductility, as the beam with hybrid rebars does not dissipate more than the beam with the steel rebars [46,47].

### 4.2. Investigation of Concrete Crushing Zone

**Figure 7:**
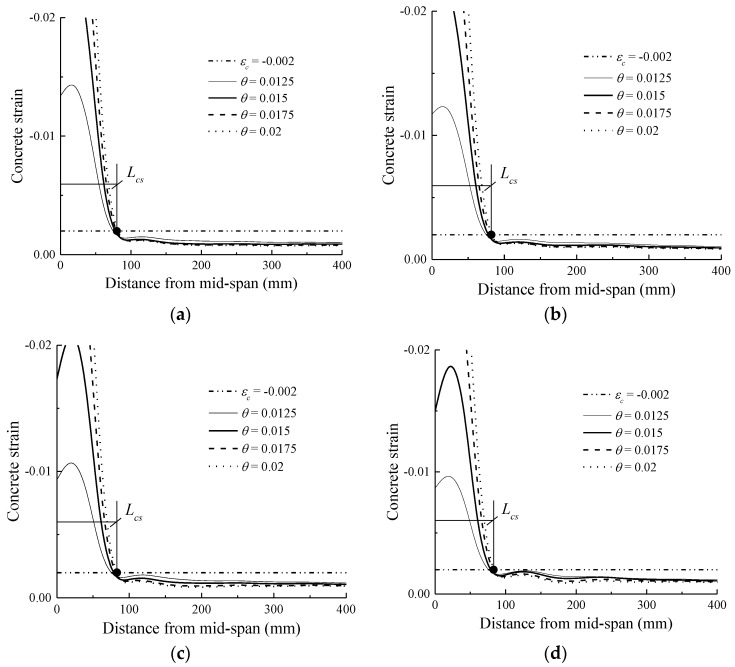
Concrete compressive strain distribution: (**a**) *ρr* = 0; (**b**) *ρr* = 0.33; (**c**) *ρr* = 1; (**d**) *ρr* = 3.

Figure 7 shows the concrete strain distribution at the extreme compression face of the beams at each drift ratio level. It can be seen from Figure 7 that an evident dividing point exists for each beam. On the left side of the dividing points, the concrete strains display an abrupt increase, whereas on the right side of the dividing point, they almost remain constant as the drift ratio increases. The vertical co-ordinate of the dividing points is approximately at the strain value of 0.002. This is because, beyond the compression strain of 0.002, concrete enters the compression softening period, and subsequently, strain localisation occurs. The simulation results are consistent with the test observations. To study the concrete crushing zone, the length of the region where the concrete compression strain is larger than the strain of 0.002 (*L_cs_*) was always selected for analysis [24,42,43].

Figure 7 also shows that the dividing points stay intact as the hybrid reinforcement ratio increases, which implies that *L_cs_* is not affected by *ρ_r_*. As *ρ_r_* increases, the concrete strength remains constant and the compression failure zone remains almost constant, resulting in a stable *L_cs_*.

### 4.3. Investigation of Curvature Localisation Zone

**Figure 8:**
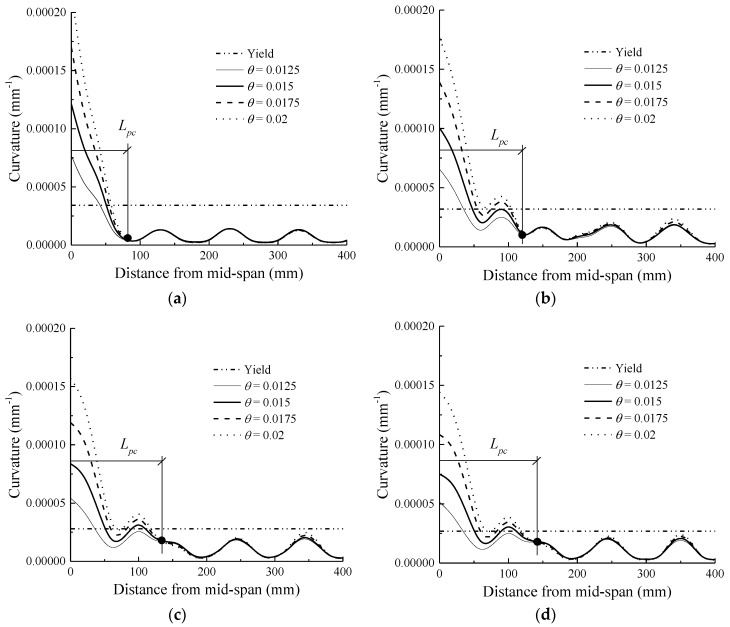
Curvature distribution along beam length: (**a**) *ρr* = 0; (**b**) *ρr* = 0.33; (**c**) *ρr* = 1; (**d**) *ρr* = 3.

Figure 8 shows the curvature distribution along the beam length for specimens at each drift ratio level. Herein, the curvature is defined by the following equation:

(11) $$\phi =\frac{\varepsilon_{t}-\varepsilon_{c}}{h}$$

where *ε_t_* and *ε_c_* are the strains at the extreme tension fibre and extreme compression fibre, respectively, and *h* is the beam depth. It can be found from Figure 8 that, similar to the rebar yielding distribution in the RC beam (Figure 5a) and the concrete compressive strain distribution (Figure 7), a dividing point exists for each beam; the curvatures fluctuate dramatically beyond yielding from this point to the mid-span section, whereas those on the left side of the dividing point remain almost constant. The simulation results are consistent with the test observations [48]. The distance from the critical section (mid-span section in this study) to the dividing point is always defined as the length of the curvature localisation zone *L_pc_* [24,42,43].

Figure 8 also shows the effect of the hybrid reinforcement ratio *ρ_r_* on *L_pc_*. It is evident that the dividing point shifts away from the mid-span section as *ρ_r_* increases, indicating a larger *L_pc_* for a higher *ρ_r_*. For example, *L_pc_* increases from 82 mm to 142 mm when *ρ_r_* changes from zero to three. It is inferred from Equation (11) that the curvature is associated with both the concrete compression strain and the tension strain. As a result, the curvature localisation is caused by concrete crushing as well as steel yielding. As *L_sy_* and *L_cs_* increase with the increase in *ρ_r_*, *L_pc_* subsequently exhibits a similar increasing trend.

At an identical drift ratio, the larger *L_pc_* at a higher *ρ_r_* is accompanied by the more uniform curvature distributions. Figure 8 clearly shows that at an equivalent mid-span displacement, a beam with a smaller *L_pc_* exhibits a larger curvature in the mid-span section, and that a larger plastic hinge length yields a smaller mid-span curvature. For example, at the drift ratio of 2%, the mid-span curvature of the beam with *ρ_r_* = 0 is 0.000219, compared with 0.000176, 0.000157, and 0.000144 for *ρ_r_* = 0.25, *ρ_r_* = 0.5, and *ρ_r_* = 0.75, respectively. This implies that at an identical displacement level, the hybrid beam with a higher hybrid reinforcement ratio exhibits a smaller mid-span curvature demand than that with a lower hybrid reinforcement ratio. Moreover, for a smaller curvature demand, the residual displacements would be smaller for the hybrid beams with a higher hybrid reinforcement ratio, owing to the self-centering behaviour of the FRP reinforcements; this results in an improved reparability of structures after a severe seismic event.

**Figure 9:**
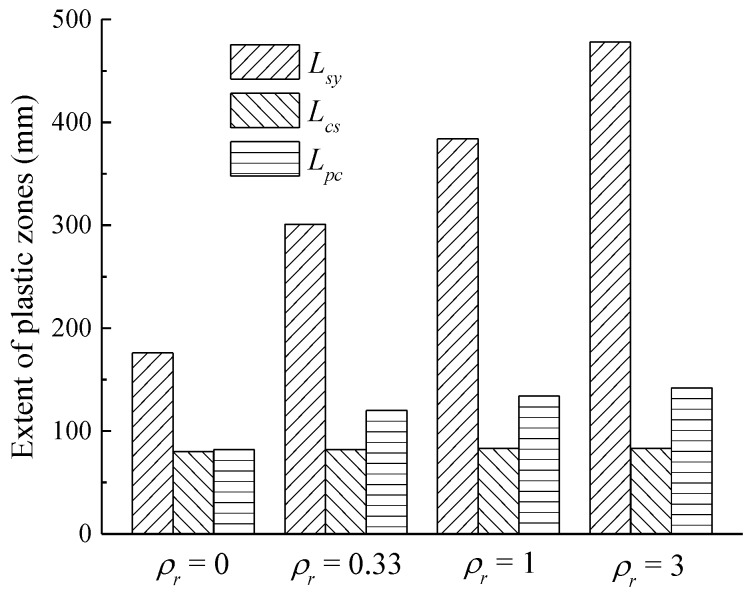
Effect of hybrid reinforcement ratio on plastic hinge length.

The influences of the hybrid reinforcement ratio *ρ_r_* on *L_sy_*, *L_cs_*, and *L_pc_* are displayed together in Figure 9. It was reported that a concrete strain of approximately 0.05–0.008 could be selected as the concrete ultimate strain [49]. Therefore, in the following sections, *L_sy_* is determined when the maximum concrete compression strain attains 0.008 for hybrid FRP–steel reinforced concrete beams. It can clearly be seen from Figure 9 that *L_cs_* remains almost constant, whereas both *L_sy_* and *L_pc_* exhibit increasing trends when *ρ_r_* increases. The growth rate of *L_sy_* is more pronounced than that of *L_pc_*.

## 5. Parametric Study

In order to gain a comprehensive understanding of the plastic hinge regions of hybrid FRP–steel reinforced concrete beams, a systematic parametric study has been carried out. The main influential parameters include the aspect ratio, *z*/*h*; concrete compressive strength, *f_co_*; yield strength of steel reinforcement, *f_y_*; steel reinforcement ratio, *ρ_s_*; steel hardening ratio, *E_sh_*; and elastic modulus of the FRP reinforcement, *E_f_*. The control beam has a steel reinforcement ratio of 0.8% and an FRP reinforcement ratio of 0.8%. The geometric dimensions and material properties of the control beam are identical to that provided in Section 4. When a factor is investigated, the other factors are maintained at a constant.

### 5.1. Effect of Aspect Ratio

**Figure 10:**
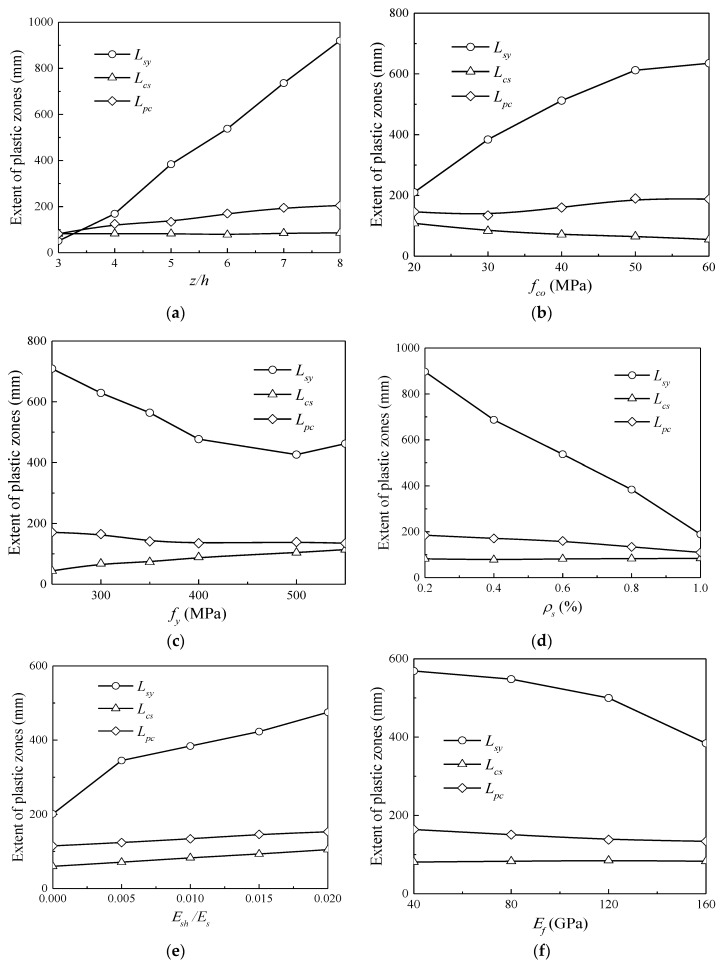
Variation of plastic hinge zones: (**a**) effect of aspect ratio; (**b**) effect of compressive strength of concrete; (**c**) effect of yield strength of steel reinforcement; (**d**) effect of steel reinforcement ratio; (**e**) effect of hardening modulus of steel reinforcement; (**f**) elastic modulus of fibre-reinforced polymer (FRP) reinforcement.

In the present study, the variation in *z*/*h* from three to eight was achieved by increasing the effective length, *z*, while fixing the beam depth, *h*, at 400 mm. Figure 10a shows the effect of the aspect ratio on the extent of the plastic hinge zones. It is evident that both *L_sy_* and *L_pc_* increase rapidly, whereas *L_cs_* remains almost constant as *z*/*h* increases. Theoretically, the distance from the maximum moment section to the yield moment section, denoted as *L_sy_*, is proportional to the beam length, as shown in Figure 6.

### 5.2. Effect of Compressive Strength of Concrete

The numerical results in Figure 10b illustrate the effect of the compressive strength of the concrete, *f_co_*, on the lengths of the plastic hinge regions of the hybrid reinforced concrete beams. *f_co_* varies from 20 MPa to 60 MPa. It is evident that *L_sy_* exhibits an increasing trend, whereas *L_cs_* exhibits a decreasing trend as *f_co_* increases. As *f_co_* increases, the compression zone is strengthened; thus, a larger tension resultant force is necessary to balance the increasing compression force, resulting in a larger maximum moment and thus a larger *L_sy_*. *L_pc_* increases marginally as *f_co_* increases, indicating that, in this case, *L_pc_* is governed by the steel yielding zone. As the ultimate curvature, which is defined by the ratio of the ultimate compression strain (0.003–0.0035) to the height of the neutral axis, keeps almost constant, a larger plastic hinge region leads to a higher ultimate deformation.

### 5.3. Effect of Yield Strength of Steel Reinforcement

The yield strength of the steel reinforcement, *f_y_*, is considered to affect the plastic hinge zones and was included in the most widely-used plastic hinge length model of the RC members, proposed by Paulay and Priestley [27]. The effect of *f_y_* on the plastic hinge lengths of the hybrid reinforced concrete beams is presented in Figure 10c. It is observed that *L_sy_* decreases, whereas *L_cs_* increases as *f_y_* increases. As *f_y_* increases, the tension zone is strengthened and the compression of concrete enters into the ultimate state, as described earlier, resulting in a smaller *L_sy_*. Meanwhile, the yield moment increases as *f_y_* increases, which implies a lower ratio of the maximum moment to the yield moment, and thus a smaller *L_sy_*. Owing to the coexisting effects of *L_sy_* and *L_cs_*, *L_pc_* remains almost constant as *f_y_* increases.

### 5.4. Effect of Steel Reinforcement Ratio

Figure 10d shows the effect of the steel reinforcement ratio, *ρ_s_*, on the extent of the plastic hinge regions for the hybrid reinforced concrete beams. The FRP reinforcement ratio is fixed to be 0.8%, whereas the steel reinforcement ratio varies from 0.2% to 1%. It can be found from Figure 10d that *L_sy_* decreases rapidly as *ρ_s_* increases, whereas *L_cs_* remains almost constant as *ρ_s_* increases. On the one hand, the tension zone of the beam is strengthened as *ρ_s_* increases, resulting in a smaller *L_sy_*. On the other hand, the larger *ρ_s_* results in a smaller gap between the maximum moment and the yield moment, which further reduces *L_sy_*. *L_pc_* is determined by *L_sy_* and thus exhibits a decreasing trend as *ρ_s_* increases.

### 5.5. Effect of Steel Hardening Modulus

The effect of the steel hardening modulus *E_sh_* on the plastic hinge lengths of the hybrid reinforced concrete beams is shown in Figure 10e. It is evident that *L_sy_*, *L_c,s_*, and *L_pc_*, exhibit increasing trends as *E_sh_*/*E_s_* increases. The mechanism is extremely straightforward and can be explained as follows: as *E_sh_* increases, the yield moment remains constant, whereas the maximum moment increases, which implies a larger extent of the rebar yielding zone, as illustrated in the moment distribution diagram in Figure 6.

### 5.6. Effect of Elastic Modulus of FRP Reinforcement

Figure 10f shows the effect of the elastic modulus of the FRP reinforcement, *E_f_*, on the plastic hinge lengths of the hybrid reinforced concrete beam. Four elastic modulus values, 40 GPa, 80 GPa, 120 GPa, and 160 GPa, have been selected for analysis. It can be seen from Figure 10f that *L_sy_* decreases as *E_f_* increases. On the one hand, as *E_f_* increases, both the yield moment and the maximum moment increase and their ratio remains almost constant, which marginally affects *L_sy_*. On the other hand, the tension zone is strengthened as *E_f_* increases, which has a negative effect on *L_sy_*. This results in a smaller *L_sy_* for a larger *E_f_*. The variation trend of *L_pc_* remains consistent with *L_sy_*, which decreases as *E_f_* increases.

## 6. Conclusions

This study examines the plastic hinge lengths of hybrid FRP–steel reinforced concrete beams, which have not been investigated extensively in previous studies. Firstly, a finite element model was proposed, and the accuracy of the model was verified by comparing the numerical results with the test results. Subsequently, three plastic hinge regions, including the rebar yielding zone, concrete crushing zone, and curvature localisation zone, were analysed in detail. The influence of the beam aspect ratio, concrete grade, steel yield strength, steel reinforcement ratio, steel hardening modulus, and FRP elastic modulus on the lengths of the three plastic zones have been studied through parametric studies. The effect of the FRP reinforcement ratio on the deformability and ductility of the hybrid reinforced concrete beams is also thoroughly discussed. The following conclusions can be drawn from the present study:

1. The variation trend in the length of the rebar yielding zone, *L_sy_*, of the hybrid reinforced beams is significantly different from that of normal RC beams. *L_sy_* continues to increase with the increase in displacement for hybrid reinforced concrete beams, whereas it is limited to a certain value for normal RC beams.
2. The hybrid reinforcement ratio exerts a significant positive effect on *L_sy_*, owing to the moment redistribution along the beam length. The curvature localisation length, *L_pc_*, increases rapidly, whereas the concrete crushing length, *L_cs_*, remains almost constant as the hybrid reinforcement ratio increases.
3. Under an identical displacement level, the hybrid beam with the larger hybrid reinforcement ratio exhibits a mid-span curvature demand and residual displacements smaller than those with a lower hybrid reinforcement ratio.
4. Among all of the parameters, *z*/*h*, *f_co_*, and *E_sh_* exert positive effects on *L_sy_* and *L_pc_*. *f_y_* and *E_sh_* exert positive effects on *L_cs_*, whereas *z*/*h*, *ρ_s_*, and *E_f_* exert negligible effects on *L_cs_*.

## Figures and Tables

**Table 1:** Details of beam specimens.

Specimen ID	*b* (mm)	*h* (mm)	*A_s_* (mm^2^)	*A_f_* (mm^2^)	*A* (mm^2^)	*f_c_* (MPa)	*f_y_* (MPa)	*E_f_* (GPa)	*A_f_* (mm^2^)
A1	150	200	100.48	88.31	188.79	45.7	465	49	1674
A2	150	200	100.48	157	257.48	45.7	465	50.1	1366
B3	180	250	226.08	253.23	479.31	23.7	363	45	782
G0.6-T1.0-A90	280	380	981.7	567.1	1448.8	44.6	550	39.5	588
